# The Main Molecular Mechanisms Underlying Methamphetamine- Induced Neurotoxicity and Implications for Pharmacological Treatment

**DOI:** 10.3389/fnmol.2018.00186

**Published:** 2018-06-04

**Authors:** Xue Yang, Yong Wang, Qiyan Li, Yaxian Zhong, Liangpei Chen, Yajun Du, Jing He, Lvshuang Liao, Kun Xiong, Chun-xia Yi, Jie Yan

**Affiliations:** ^1^Department of Forensic Science, School of Basic Medical Sciences, Central South University, Changsha, China; ^2^Department of Anatomy and Neurobiology, School of Basic Medical Science, Central South University, Changsha, China; ^3^Department of Endocrinology and Metabolism, Academic Medical Center, University of Amsterdam, Amsterdam, Netherlands

**Keywords:** methamphetamine, neurotoxicity, oxidative stress, excitotoxicity, neuroinflammation, immunotherapy

## Abstract

Methamphetamine (METH) is a popular new-type psychostimulant drug with complicated neurotoxicity. In spite of mounting evidence on METH-induced damage of neural cell, the accurate mechanism of toxic effect of the drug on central nervous system (CNS) has not yet been completely deciphered. Besides, effective treatment strategies toward METH neurotoxicity remain scarce and more efficacious drugs are to be developed. In this review, we summarize cellular and molecular bases that might contribute to METH-elicited neurotoxicity, which mainly include oxidative stress, excitotoxicity, and neuroinflammation. We also discuss some drugs that protect neural cells suffering from METH-induced neurotoxic consequences. We hope more in-depth investigations of exact details that how METH produces toxicity in CNS could be carried out in future and the development of new drugs as natural compounds and immunotherapies, including clinic trials, are expected.

## Introduction

Methamphetamine (METH), also known as “ice” or “crystal,” is an addictive pharmacologic psychostimulant with strong neurotoxic effects on the central nervous system (CNS). It has been abused by >33 million people worldwide and seen a steady increase in use over the last few decades. Such use is associated with deleterious effects on families, loss of productivity, major public-health concerns, and a consumption of substantial resources for medical intervention (Courtney and Ray, 2014; Moratalla et al., 2017). A recent editorial in the *Lancet* stated that the shift in public-health priorities to opioids in the last few years in the United States has enabled the METH market to flourish; as a result, this market is primed for resurgence. Accordingly, drug control may be more challenging than anticipated as a second “METH wave” begins (Lancet, 2018).

Methamphetamine belongs to a class of synthetic drugs known as amphetamine-type stimulants, which includes amphetamine, METH, methylenedioxy-methamphetamine, and other designer drugs (Chomchai and Chomchai, 2015). METH is similar to amphetamine with regard to pharmacodynamic effects; however, users are more likely to become addicted to METH because of its better penetration into the CNS and longer duration of action (Won et al., 2013). Long-term abuse of METH causes serious physical and mental damage. Overall, METH abuse is associated with an increased risk of infection by the human immunodeficiency virus, hepatitis viruses, as well as dangerously high body temperature, periodontal disease, pulmonary hypertension, adrenergic storm, cerebrovascular events, stroke, circulatory collapse, and kidney failure (Ho et al., 2009; Schep et al., 2010; Moratalla et al., 2017). METH abusers are more likely to develop Parkinson’s disease, depression, schizophrenia, psychosis, and other neuropsychiatric and cognitive sequelae (Rawson and Condon, 2007; Forray and Sofuoglu, 2014; Hsieh et al., 2014); these are mostly attributed to METH-induced neurotoxicity. The neurotoxic effects of METH are of strong concern, and exploration of the mechanisms underlying this neurotoxicity has become a research hotspot in recent years (Xiong et al., 2016; Ashok et al., 2017; Xie et al., 2018).

In general, neurotoxicity is defined as physical damage to neurons. In a broader sense, neurotoxicity may refer to a permanent or reversible adverse effect of a substance on neuronal structure/function that induces disruption of neuronal components, collapse of entire neurons, histologic signs of neuronal injury, and/or behavioral abnormalities (Moszczynska and Callan, 2017). METH-induced neurotoxic effects include damage to dopaminergic and serotonergic terminals, neuronal apoptosis, as well as activated astroglia and microglia that lead to a neuroinflammatory response within the brain (Cadet and Krasnova, 2009; Panenka et al., 2013; Moratalla et al., 2017).

In clinical trials, psychological therapies have been shown to generate small-to-moderate reductions in METH use; however, these have not yet been translated into clinical practice (Colfax et al., 2010; Carroll, 2014). Poor outcomes of psychosocial interventions may be related to METH-produced neurotoxicity. Further, psychosocial treatments must be utilized clinically in conjunction with other strategies as pharmacotherapies (Aharonovich et al., 2006).

In this review, we discuss briefly some of the principal mechanisms underlying the neurotoxicity induced by METH and summarize targeted pharmacologic treatments. We anticipate that more efficacious intervention strategies that protect neural cells against METH-induced neurotoxic consequences may be implemented in the future.

## Mechanisms Underlying METH-Induced Neurotoxicity

### Oxidative Stress

**FIGURE 1:**
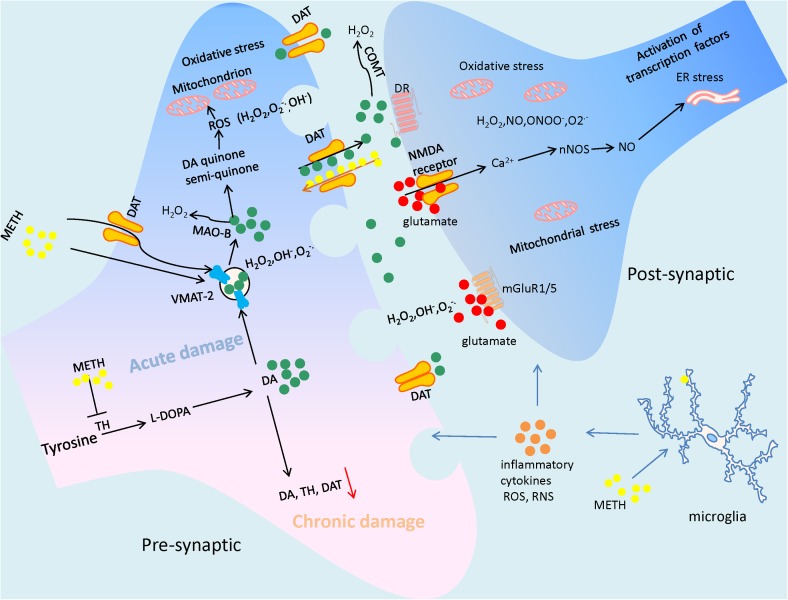
The illustration summarizes the main mechanisms of Methamphetamine (METH)-elicited neurotoxic effects, which include DA oxidation, excessive glutamate production, generating a large amount of reactive oxygen species (ROS) and reactive nitrogen species (RNS), and subsequently leading to mitochondrial dysfunction and ER stress. The neuroinflammation mediated by microglial cells also contribute to the neuronal damage by attacking it with inflammatory cytokines. As a result of the suffering from METH, the neuronal cells may undergo terminal degeneration or apoptosis. In particular, due to the neurotoxicity of the drug, long time abuse of METH often cause the decrease of dopaminergic markers such as dopamine (DA), tyrosine hydroxylase (TH), and dopamine transporter (DAT).

The neurotoxic mechanism of METH is complex and involves multiple pathways. Oxidative stress has been demonstrated to be a significant factor contributing to cellular toxicity. METH induces the considerable production of reactive oxygen species (ROS), such as hydroxyl radicals (OH^-^), hydrogen peroxide (H_2_O_2_), and the superoxide anion (O_2_^-^), by increasing the oxidation of dopamine (DA) (Hansen, 2002). METH passes through the blood–brain barrier and penetrates the brain readily due to its high lipid solubility (Nordahl et al., 2003). Then, it enters dopaminergic terminals via the dopamine transporter (DAT) because of its similarity to DA (Shin et al., 2017), as well as by passive diffusion (Moszczynska and Callan, 2017). METH enhances DA concentration in the cytosol and synaptic cleft significantly by impairing vesicle monoamine transporter 2 (VMAT2) function and promoting DA release; this process may represent the main mechanism underlying the neurotoxic effect of METH in the brain (Baumann et al., 2002). Within dopaminergic terminals and in synaptic clefts, excess DA is autoxidized to quinone or semi-quinone (LaVoie and Hastings, 1999) to generate large amounts of H_2_O_2_, OH^-^, and O_2_^-^ (Baumann et al., 2002). Further, a small proportion of DA metabolism mediated by monoamine oxidase (MAO) or catechol-*O*-methyltransferase (COMT) produces H_2_O_2_ as a byproduct (Cadet and Brannock, 1998; Olanow and Tatton, 1999). H_2_O_2_ reacts with transition-metal ions to produce highly toxic OH^-^. Eventually, abundant ROS promote a series of oxidative stress reactions, such as lipid peroxidation and activation of proteases, which trigger the cell-death cascade. Furthermore, highly toxic peroxynitrite ions (ONOO^-^) produced via O_2_^-^ react with nitric oxide (NO) to damage proteins, nucleic acids, and phospholipids in cells by circumventing antioxidative enzymes (Cadet and Brannock, 1998; **Figure 1**).

Mitochondria represent a major site of METH-induced ROS production within neural cells (Dawson and Dawson, 2017). Mitochondria, which are intracellular organelles composed of two bilayers, act as the energy generators of cells through oxidative phosphorylation and adenosine triphosphate (ATP) production. Defects in mitochondrial respiration have been implicated in neuronal death and several neurodegenerative diseases (Dawson and Dawson, 2017). Several studies have suggested that dysfunction of mitochondrial metabolism plays a critical role in METH-induced dopaminergic neurotoxicity through inhibition of the Krebs cycle and electron transport chain (ETC) as well as by promotion of oxidative stress; these effects result in imbalance between oxidation and antioxidation in neural cells (Annepu and Ravindranath, 2000; Beer et al., 2004; Shin et al., 2017). ROS and reactive nitrogen species (RNS) generated by DA oxidation inhibit several key enzymes directly as complexes I, II, III, and IV of the ETC, causing mitochondrial dysfunction and damage to DNA structure as well as loss of genetic information (Burrows et al., 2000; Brown et al., 2005; Bachmann et al., 2009; Moratalla et al., 2017; Moszczynska and Callan, 2017). In turn, inhibition of ETC components by METH enhances O_2_^-^ production due to electron leakage. This positive-feedback loop aggravates disturbance of mitochondrial energy metabolism and neurotoxicity.

The changes in mitochondrial enzymes in response to METH remain controversial. Klongpanichapak et al. (2006) found significant inhibition of expression of striatal complex I following repeated METH treatment in mice. In accordance with this finding, Thrash et al. (2010) and Thrash-Williams et al. (2013) showed that intraperitoneal administration of METH decreased the activity of striatal complex I significantly but had no significant effect on the activity of complex IV *in vivo* or *in vitro*. Several researchers have found that acute exposure to METH induces a decrease in glutathione (GSH) levels and an increase in levels of oxidized glutathione (GSSG) in the striatum; this leads to a reduction in the GSH/GSSG ratio, which is essential for the inhibition of striatal complex I activity (Annepu and Ravindranath, 2000; Beer et al., 2004). In contrast, a regimen of rapid (<1 h) binge administration of METH decreased complex II activity in the striatal brain regions, but did not decrease the activity of complex I *in vivo* (Brown et al., 2005). Killinger et al. (2014) also found that a binge regimen of METH every 2 h, via four successive intraperitoneal injections, did not alter the levels of mitochondrial complex I in striatal synaptosomes *in vivo*. An *in vitro* study showed no significant alterations in the protein content of mitochondrial respiratory complex I; however, METH treatment caused time-dependent reductions in the protein contents of complex IV (Wu et al., 2007). Although the reactions of the mitochondrial ETC differ according to the route of drug delivery and modes of METH administration, these studies suggest that METH inhibits the activity of the mitochondrial respiratory chain complex; this effect is considered to play a crucial role in the pathogenesis of several psychiatric disorders such as depression, bipolar disorder and schizophrenia (Manji et al., 2012).

In recent years, dynamic disorders of mitochondria have been reported to result from mitochondrial dysfunction triggered by METH (Lenzi et al., 2012; Lin et al., 2012). In general, mitochondrial biogenesis coupled with dynamic fusion and fission maintain healthy mitochondria, whereas damaged mitochondria are degraded by mitophagy (Michel et al., 2012). The key molecules of mitochondrial biogenesis are peroxisome proliferator-activated receptor gamma coactivator-1α (PGC-1α), nuclear respiratory factors (NRFs), and mitochondrial transcription factor A (TFAM) (Lee and Wei, 2005). PGC-1α regulates and coordinates the activity of NRF and TFAM to serve as a nutrient-sensing system that increases mitochondrial biogenesis (Nervina et al., 2006). Using a repeated escalating METH regimen in rats, Valian et al. (2017) detected an increase in expression of PGC1α and TFAM in the substantia nigra, but not the striatum. However, Beirami et al. (2018) revealed a significant decrease in expression of PGC-1α, NRF, and TFAM in the hippocampus of rats with a different repeated METH administration regimen. These inconsistent results suggest that the indicators of mitochondrial biogenesis appear to be expressed aberrantly in various brain regions or according to the route of drug administration.

The regulation of mitochondrial fusion is primarily through mitofusin-1, mitofusin-2, and optic atrophy protein 1 (Opa1), whereas mitochondrial fission is regulated by mitochondrial fission 1 protein (Fis1) and dynamin-related protein 1 (Drp1) (Ding et al., 2012). In *in vitro* studies, Parameyong et al. (2013, 2015) revealed that METH decreased cell viability significantly and increased the levels of Fis1 and Drp1 in isolated mitochondria, whereas Drp1 expression in the cytosol of METH-treated cells showed no significant differences compared with the control group. Interestingly, Borgmann and Ghorpade (2018) reported a larger and more rod-shaped morphology and dysfunction of mitochondria in astrocytes during prolonged exposure to low levels of METH. This may have been mediated by inhibition of phosphorylation of Drp1 and an increase in mitofusin levels, implying an overall increase in the number of mitochondria in astrocytes (Borgmann and Ghorpade, 2018). Considering the difference of dose and time points of METH used in these studies, the regimen of drug administration may be the main cause of the alteration in mitochondrial dynamics. Nonetheless, the mechanisms of mitochondrial impairment induced by METH have not been elucidated to date.

Oxidative stress occurs primarily in mitochondria and leads to mitochondrial dysfunction by attacking mitochondria in the CNS (Thrash-Williams et al., 2016). Accordingly, METH-induced mitochondrial damage may contribute to dopaminergic toxicity by enhancing susceptibility to oxidative stress and promoting the apoptosis of neural cells. This phenomenon is of clinical relevance as it eventually results in devastating neuropathological effects in the brain due to mitochondrial impairment, subsequent caspase activation, and apoptotic neuronal death following METH administration (Nguyen et al., 2015; Chamorro et al., 2016; Xiong et al., 2017). For instance, METH exposure has been shown to increase expression of the pro-apoptotic proteins Bax, Bad, and Bid (Jayanthi et al., 2001; Bachmann et al., 2009; Beauvais et al., 2011; Raineri et al., 2012) and decrease the expression of the anti-apoptotic proteins Bcl-2 and Bcl-xL (Jayanthi et al., 2001; Beauvais et al., 2011; Raineri et al., 2012). The decrease in the Bcl-2/Bax ratio in mitochondrial fractions has been shown to promote cytochrome c (Cyt c) release from mitochondria (Qiao et al., 2014; Nam et al., 2015). Subsequently, Cyt c becomes part of the apoptosome with apoptotic peptidase activating factor-1 (Apaf-1) and induced sequential activation of the apoptosis executioners caspase-3, -6, and -7 (Shin et al., 2017).

Several studies have reported that multiple molecules are involved in the apoptotic death of neurons induced by METH. For instance, Kim et al. (1999) found that inhibition of protein kinase C delta (*PKC*δ) or overexpression of a cleavage-resistant *PKC*δ mutant protected against METH-elicited apoptosis in mesencephalic dopaminergic cell cultures *in vitro* (Lin et al., 2012). In follow-up studies, they showed that *PKC*δ inhibition may rescue METH-elicited mitochondrial burden, pro-apoptosis, and dopaminergic degeneration, implying that *PKC*δ is an important gene involved in METH-induced mitochondrial dysfunction and apoptosis in dopaminergic neuronal cells (Nam et al., 2015; Nguyen et al., 2015; Shin et al., 2016; Mai et al., 2018).

**FIGURE 2:**
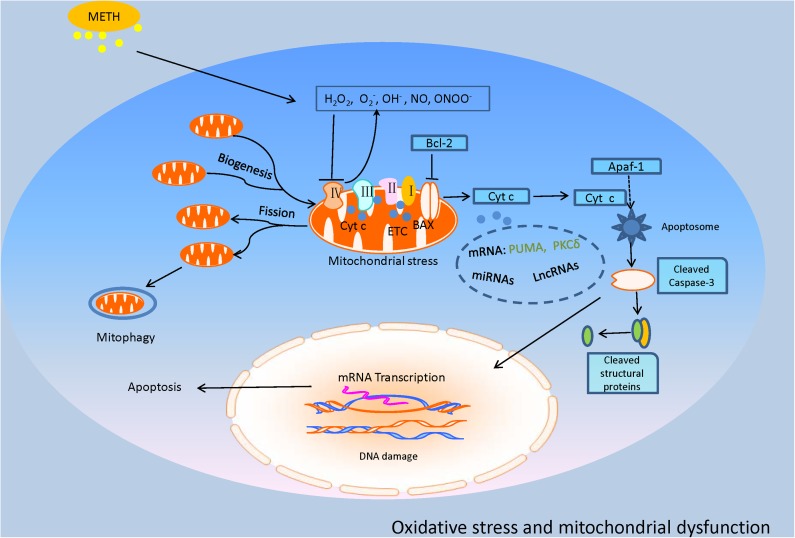
This model illustrates the oxidative stress and mitochondrial dysfunction involved in METH-induced neurotoxic consequences. METH exposure produced a considerable amount of ROS and RNS, named OH^-^, H_2_O_2_, O_2_^-^, NO, and ONOO^-^. The excessive oxidative stress inhibits the key enzymes of the ETC, causing mitochondrial dysfunction that leads to mitochondrial fission and mitophagy. Particularly, the impaired mitochondria trigger the increase of Bax and decrease of Bcl-2 and sequential cytochrome c (Cyt c) release, inducing activation of executioner caspases-3 and apoptosis which might be regulated by some molecules such as p53-upregulated modulator of apoptosis (PUMA), protein kinase C delta (PKCδ), miRNAs, and long non-coding RNAs (lncRNAs) which are reviewed in the text.

Recently, Chen et al. (2016) reported that p53-upregulated modulator of apoptosis (PUMA) was involved in the mitochondrial apoptotic pathway induced by METH in PC12 cells and SH-SY5Y cells. They suggested that PUMA interacts with Bax and Bcl-2 in mitochondrial membranes to drive Cyt c relocation from mitochondria to the cytoplasm, causing activation of caspase-3, poly-ADP-ribose polymerase (PARP) and apoptosis (Chen et al., 2016). Furthermore, through microinjection of anti-micro (mi)R143 into the hippocampi of mice, Zhang et al. (2016) revealed that miR143-dependent PUMA upregulation reversed the METH-induced decrease in microglial survival via regulation of apoptosis and autophagy. In addition to miRNAs, long non-coding RNAs (lncRNAs) appear to participate in METH-induced neuronal apoptosis by regulating the coding genes of neurons. In our recent investigation, we reported that several lncRNAs were expressed differentially in primary cultured prefrontal cortical neurons treated with METH. Further, using bioinformatics, we hypothesized that lncRNA GAS5 modulates downstream molecules involved in p53-mediated neuronal apoptosis, although more direct evidence from *in vivo* and *in vitro* studies is needed (**Figure 2**; Xiong et al., 2017).

### Excitotoxicity

Glutamate (Glu), which is the main excitatory neurotransmitter in the brain, has been reported to have an important role in METH-induced excitotoxicity (Moratalla et al., 2017). Glu accumulation over-activates various downstream signaling pathways, mostly involving a surge in Ca^2+^ influx, to trigger an increase in intracellular Ca^2+^ concentration (Chamorro et al., 2016). Specifically, excessive Glu activates *N*-methyl-D-aspartate receptors (NMDARs) and metabotropic glutamate receptors (mGluRs) (Tseng et al., 2010). Activation of mGluRs induces protein kinase C (PKC) phosphorylation and upregulates NMDAR function, leading to an increase in Ca^2+^ influx, which acts as a pervasive and pluripotent second messenger (Bahar et al., 2016). Excess intracellular Ca^2+^ triggers a cascade of reactions within cells to activate protein kinases, phosphatases, and nitric oxide synthase (NOS). The latter subsequently promotes NO production (Tseng et al., 2010), which causes endoplasmic reticulum (ER) stress, activation of apoptotic pathways and, eventually, METH-produced neurotoxic sequelae (Moratalla et al., 2017).

**FIGURE 3:**
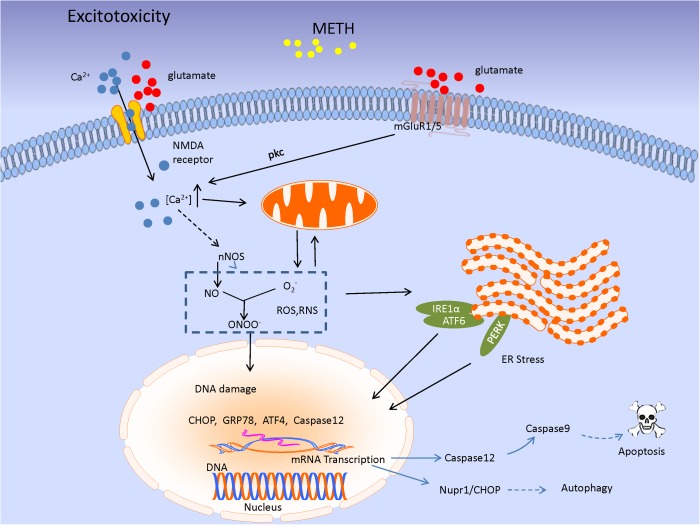
The image shows the excitatory toxicity model of METH. METH-mediated increase in extracellular glutamate level leads to stimulation of mGluR1/5 or *N*-methyl-D-aspartate receptors (NMDARs). mGluR1/5-induced protein kinase C (PKC) activation phosphorylates and upregulates NMDAR function, leading to Ca^2+^ influx. The signaling results in enhancement of cytosolic Ca^2+^ level associated with nNOS activity, leading to NO production. NO acts as an ER stressor, and then, UPR signaling pathway would be initiated in response to ER stress through three ER transmembrane mediators [inositol requiring protein-1 (IRE1)α, activating transcription factor-6 (ATF6), and protein kinase RNA-like ER kinase (PERK)]. Subsequently, the mediators lead to special genes transcription as CHOP, GRP78, and Caspase 12 which triggering a series of cascade involving apoptosis and autophagy.

In particular, ER stress occurs under various toxic stimuli along with accumulation of misfolded proteins and activation of unfolded protein response (UPR), which removes unfolded and/or misfolded proteins in the ER, thereby recovering ER homeostasis. ER stress leads to the activation of three ER-resident transmembrane proteins: activating transcription factor-6 (ATF6), inositol requiring protein-1 (IRE1) and protein kinase RNA-like ER kinase (PERK) (Szegezdi et al., 2006; Hetz, 2012). The roles of these three signaling pathways involve a reduction in protein synthesis and expression of specific genes to cope with proteotoxic stress (Wongprayoon and Govitrapong, 2017). During prolonged ER stress, IRE1, PERK, and ATF6 may induce pro-apoptotic signaling through activation of C/EBP homologous protein (CHOP), which subsequently leads to the initiation of ER stress-mediated apoptosis through regulation of Bcl-2 family members (Bahar et al., 2016). ER stress leads to apoptosis, including various mechanisms involving activation of death receptors and participation of the mitochondria-dependent cell death pathway (Sano and Reed, 2013). It has been shown that neurotoxic doses of METH induce expression of several ER stress genes, including those that encode the 78-kDa glucose-regulated protein (GRP-78), CHOP, and ATF4, in the rat striatum (Jayanthi et al., 2004; Beauvais et al., 2011). The ER stress induced by METH appears to be associated with dopaminergic toxicity and activation of the DA D1 receptor (Beauvais et al., 2011). Recently, Wongprayoon and Govitrapong (2017) suggested that METH-induced apoptotic death is mediated (at least in part) through an ER-dependent mechanism involving CHOP, spliced X-box binding protein 1 (XBP1), caspase-12, and caspase-3 *in vitro*. In addition, exposure to relatively high-dose METH increases nuclear protein 1 (Nupr1) expression, which promotes dopaminergic neuronal apoptosis and autophagy through a Nupr1/CHOP pathway (**Figure 3**; Xu X. et al., 2017).

### Neuroinflammation

Reactive neurogliocytes are considered to be sensitive markers of nerve damage, which is a common response to CNS injury (Ares-Santos et al., 2013). The neuroinflammation caused by METH shows a close correlation with microglial activity as it is activated rapidly after METH administration in DA-innervated areas. Microglial activation following METH exposure may result from the neuronal release of damage-associated molecular patterns (DAMPs) (Xu E. et al., 2017). For instance, high-mobility group box-1 (HMGB1) expression was found up-regulated in response to METH treatment and shown to mediate the neuroin?ammatory response in the nucleus accumbens, ventral tegmental, and prefrontal cortex of the brain (Frank et al., 2016). Thomas and colleagues suggested that METH-induced microglial activation was regulated by DA-quinone (DAQ), a metabolite of DA, because it activated microglia dose- and time-dependently, and because inhibition of DAQ formation blocked (at least partially) microgliosis (Thomas et al., 2008). The vital role of DA in neuroinflammation was also evidenced by the observation that excessive DA released into the synaptic cleft may stimulate regional microglia directly and trigger a neurotoxic signal cascade (Thomas et al., 2008). The underlying mechanism of microglial activation induced by METH is related to the Toll-like receptor 4 (TLR4) located on microglia, which is involved in the immune surveillance of pathogens and exogenous small molecules (Bachtell et al., 2015). Activation of microglia may also be mediated through the sigma-1 receptor, which involves ROS generation and activation of the mitogen-activated protein kinase (MAPK) and PI3K/Akt pathways in the neurotoxicity of METH (Chao et al., 2017).

**FIGURE 4:**
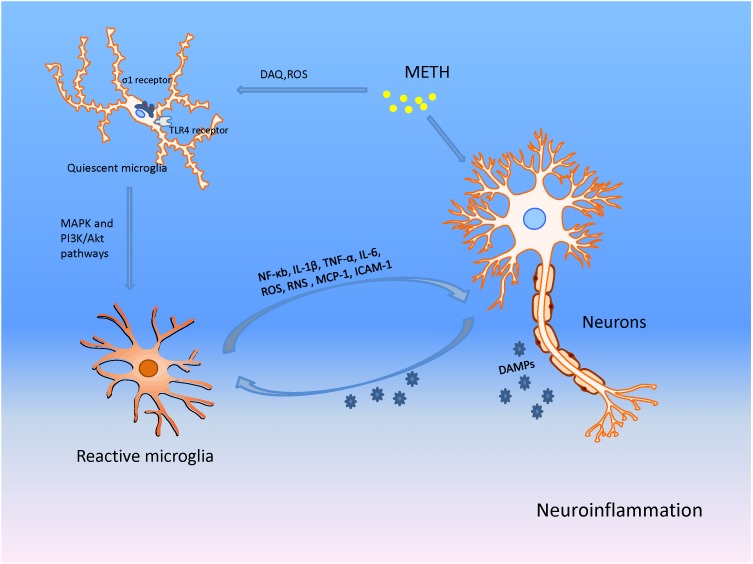
METH produces neuronal damage through microglia associated neuroinflammation in addition to direct actions on neurons. METH damages presynaptic terminals of neurons causing the production of DA-quinone (DAQ) and sequential ROS; these facilitate microglial activation. The activated microglia then increase production of nuclear factor-kappa B (NF-κB), tumor necrosis factor-α (TNF-α), interleukin 6 (IL-6), interleukin 1β (IL-1β), monocyte chemo-attractant protein 1 (MCP-1), ICAM-1, ROS, and RNS, promoting neuroinflammation and neuronal injury. The damaged neurons release DAMPs that act on microglia, and aggravate the inflammation and eventual neurotoxicity through the positive feedback mechanism.

Activated microglial cells secrete not only neurotrophic factors to prolong neuronal survival, but also cytotoxic mediators and cytokines that induce inflammation and neurotoxicity (Zhang et al., 2016). Studies have suggested that METH activates nuclear factor-kappa B (NF-κB), inducing its transfer to the nucleus and promoting the transcription of pro-inflammatory cytokines in microglia (Ojaniemi et al., 2003; Shah et al., 2012; Snider et al., 2013). This results in the release of various pro-inflammatory factors such as interleukin 6 (IL-6), interleukin 1β (IL-1β), tumor necrosis factor-α (TNF-α), monocyte chemo-attractant protein 1 (MCP-1), and cellular adhesion molecule (ICAM-1) (Yamaguchi et al., 1991; Nakajima et al., 2004; Goncalves et al., 2008; Snider et al., 2012), which are thought to play key roles in METH-induced neuroinflammation (**Figure 4**). Interestingly, astrocytes (which protect neurons and promote sprouting) are activated because glial fibrillary acidic protein (GFAP) immunoreactivity increases in the striatum and indusium griseum upon METH treatment, thereby implying complex mechanisms involving guidance molecules and cytotoxic mediators in the neurotoxic consequences of METH (Ares-Santos et al., 2013; Moratalla et al., 2017).

### Long-Term Neurotoxicity

The effects of long-term exposure to METH are different from those of acute injury. The former is characterized by impairment of expression of tyrosine, tyrosine hydroxylase (TH), DAT, and serotonin transporters (SERT), as well as DA depletion, decrease in the density of DA D2 receptors, hypo-dopaminergic status, and neuronal degeneration (Barr et al., 2002). In Wilson et al. (1996) observed decreased expression of DA, TH, and DAT in the striatum of chronic METH abusers *post mortem*, which might explain the dysphoric effects of the drug and dose escalation observed in some METH users (Wilson et al., 1996). The decrease in levels of dopaminergic markers in the striatum was shown by other studies, and could last for months to years after METH abstinence in human abusers of METH (Volkow et al., 2001a,b; Kitamura et al., 2007). Besides the striatum, a long-lasting decrease in DAT levels was also observed in the nucleus accumbens and prefrontal cortex of the brain (Davidson et al., 2001; Sekine et al., 2001). In accordance with this study, several previous reports have demonstrated that the density of DAT, VMAT2, SERT, and DA D2 receptors is significantly lower than that in healthy controls according to positron emission tomography (PET) scans of the brains of METH abusers (Sekine et al., 2001; Volkow et al., 2001b, 2015; Kish et al., 2009; Boileau et al., 2016). Similar to that observed in METH abusers, significant and sustained reductions in the levels of DA, TH, and DAT were shown following single, high-dose, or binge administration of METH in animal models (**Figure 1**; Hassan et al., 1973; McConnell et al., 2015; Nguyen et al., 2015). These results suggest persistent DA deficits as well as structural and metabolic dysfunctions in specific brain regions that correlate with several types of behavioral neurologic sequelae induced by METH (Moszczynska and Callan, 2017).

When using a regimen that involves gradual increases in METH administration to rats to mimic progressively larger doses of the drug used by some human abusers of METH, METH preconditioning protects against DA depletion caused by binge METH challenge in the brain (Cadet et al., 2011; Shen et al., 2016; Li et al., 2017). Further, chronic injections of METH activate programs that prevent DA toxicity without influencing drug-induced pathologic changes in serotoninergic systems (Cadet et al., 2009). The plethora of exciting applications of preconditioning in neuroprotection (Shen et al., 2016; Li et al., 2017) reflect differences in the mechanisms involved in METH toxicity on monoaminergic systems between the various routes of METH administration. For instance, Cadet et al. (2011) reported differential METH-induced gene expression in the striatum, including brain-derived neurotrophic factor (BDNF), heme oxygenase-1 (HO-1) and heat shock protein 27 (Hsp27), in the absence and presence of METH preconditioning and between various brain regions. This result implies that the brain cannot be considered as a homogeneous structure when assessing the molecular effects of METH preconditioning (Cadet et al., 2011; **Table 1**).

**Table 1:** The summary of common METH treatment protocols *in vivo* or *in vitro*.

Species	METH dosing regimen	Key results	Reference
SH-SY5Y cells	1.0 mM for 24 h	Cell death and mitochondrial dysfunction	Parameyong et al., 2013, 2015
SH-SY5Y cells	1.5 mM for 12 h	Mitochondrial and oxidative damage	Nam et al., 2015
SH-SY5Y cells	1.5 mM for 24 h	ER stress and cell apoptosis	Wongprayoon and Govitrapong, 2017
SH-SY5Y cells	1.68 mM for 24 and 48 h	Oxidative stress and cell death	Wu et al., 2007
SH-SY5Y cells	2.0 mM for 24 h	Neuron apoptosis	Chen et al., 2016
PC12 cells	3.0 mM for 24 h	Neuronal apoptosis and autophagy	Qiao et al., 2014; Chen et al., 2016; Xu X. et al., 2017
Astrocytic cells	500 μM for 24 h	Neuroinflammation	Shah et al., 2012
Human astrocytes	50 nM, 5 μM, 100 μM, or 500 μM, for 24 h to 16 days	Oxidative stress	Borgmann and Ghorpade, 2018
Primary cultures of rats embryonic cortical neurons	4.0 mM treatment for 24 h	Necroptosis	Xiong et al., 2016
Rat mesencephalic Dopaminergic neuronal cell line (N27 cells)	2 mM for 24 h or 0.5 mM for 1 week	Autophagy	Lin et al., 2012
Mice	A single dose of 3 mg/kg i.p.	Hypermotor activity	Snider et al., 2012
Mice	A single dose of 30 mg/kg i.p.	Neuroinflammation	Goncalves et al., 2008
Mice	A single dose of 40 mg/kg	Cell apoptosis	Jayanthi et al., 2001
Mice	1 mg/kg s.c., every day for 7 days	Cognitive deficits	Hsieh et al., 2014
Mice	2 mg/kg i.p. ×4, at 24 h intervals	Memory impairment	Hsieh et al., 2014
Mice	4 mg/kg i.p. ×4, at 2 h intervals	Drug dependence, extracellular DA release	Nakajima et al., 2004
Mice	4, 6, or 8 mg/kg i.p. ×4, at 2 h intervals	Hyperthermia, hypoactivity, activated striatal glia	McConnell et al., 2015
Mice	5 mg/kg i.p. ×4, at 2 h intervals	Neuroinflammation, microglial activation	Thomas et al., 2008; Raineri et al., 2012
Mice	8 mg/kg i.p. ×4, at 2 h intervals	Dopaminergic deficit	Nam et al., 2015
Mice	10 or 20 mg/kg ×2, at 2 h intervals	Oxidative stress	Thrash et al., 2010
Mice	10 mg/kg i.p. ×4, at 2 h intervals	Oxidative stress	Zhang et al., 2012
Mice	10 mg/kg i.p. ×2, at 2 h intervals	Dopamine depletion, excitotoxicity	Thrash-Williams et al., 2013
Mice	10 mg/kg i.p.×2, at 2 h intervals	Oxidative stress, mitochondrial dysfunction	Thrash-Williams et al., 2016
Mice	15 mg/kg i.p. every day for 7 days	Dopaminergic markers decreases	Klongpanichapak et al., 2006
Mice	30 mg/kg i.p. ×4, at 2 h intervals	Microglial activation	Chao et al., 2017
Mice	60 mg/kg, i.p. twice a day for four consecutive days	Oxidative stress	Shin et al., 2016
Rats	A single dose of 10 mg/kg i.p.	Neuroinflammation	Frank et al., 2016
Rats	0.001, 0.03, 0.1 mg/kg/day self-administration for three consecutive days	Microglial activation, neuroinflammation	Snider et al., 2013
Rats	0.3 and 1.0 mg/kg i.v. for 2 weeks	METH addiction	Baumann et al., 2002
Rats	2.5 mg/kg s.c., twice per day, for 7 days	Decreased GABA, glutamate, and glutamine levels in the PFC	Hsieh et al., 2014
Rats	5 mg/kg i.p. ×6, at 1 h intervals	Dopamine depletion	Cadet et al., 2009
Rats	10 mg/kg i.p. ×4, at 2 h intervals	Mitochondrial dysfunction	Burrows et al., 2000; Brown et al., 2005
Rats	10 mg/kg i.p. ×4, at 2 h intervals	Striatal ER and mitochondrial stress pathways	Beauvais et al., 2011
Rats	15 mg/kg i.p. ×8, at 12 h intervals	Hepatic injury, oxidative stress, cell autophagy and apoptosis	Xie et al., 2018
Rats	15 mg/kg i.p. ×4, at 2 h intervals	Monoaminergic terminal loss	LaVoie and Hastings, 1999
Rats	15 mg/kg i.p. ×8, at 12 h intervals	Neuronal apoptosis and autophagy	Xu X. et al., 2017
Rats	Repeated escalating doses: 1–14 mg/kg i.p., twice a day, at 6 h intervals, for 14 days	Dopaminergic neurons deficits	Valian et al., 2017
Rats	Repeated escalating doses: 1–10 mg/kg, twice a day, at 5 h intervals, for 10 days	Cognitive deficits	Beirami et al., 2018
Monkeys	2 mg/kg i.m. ×4, at 2 h intervals	Oxidative stress	Hashimoto et al., 2004


*i.p., intraperitoneal; i.v., intravenous; s.c., subcutaneous; i.m., intramuscular; ER, endoplasmic reticulum; METH, Methamphetamine.*

## Treatment of METH-Induced Neurotoxicity

### Targeting Oxidative Stress and Mitochondrial Toxicity

Preclinical and clinical investigations have been applied to seek effective and efficacious pharmacologic strategies for treatment of METH neurotoxicity. As stated above, METH interferes with DA reuptake and leads to DA oxidation; this leads to the production of ROS and RNS, which trigger degeneration of dopaminergic terminals and neuronal apoptosis. This pathway demonstrates that oxidative stress is one of the main mechanisms through which METH injures the CNS. Accordingly, antioxidant pharmacotherapies have been applied to explore efficacious strategies for the protection of neural cells suffering from oxidative stress generated by METH.

For example, vitamin C (Vit.C) reduces the production of free radicals, maintains GSH homeostasis, and induces the expression of HO-1, which is critical in limiting cellular damage by maintaining redox homeostasis within the brain (Rice, 2000; Hediger, 2002). Huang et al. (2012) found that pretreatment with Vit.C enhanced METH-elicited HO-1 expression and attenuated METH-induced ROS production in neuronal/glial co-cultures. Conversely, pharmacologic inhibition of HO-1 activity abolished the suppressive effects of Vit.C (Huang et al., 2012). Recently, Huang et al. (2017) found that treating cells with Vit.C before METH exposure attenuated production of ROS and Beclin 1 time-dependently, suggesting that the protective effect of Vit.C against METH toxicity is achieved via the attenuation of ROS production and apoptosis, and that HO-1 induction by Vit.C serves as a strategy for alleviation of METH neurotoxicity. However, an *in vivo* study to test the neuroprotective action of Vit.C in terms of METH exposure has not been conducted.

Selenium is an essential mineral found naturally in water, soil, and food. It is often used as an antioxidant and dietary supplement (Wang et al., 2017). In an *in vivo* study, Kim et al. (1999) found that selenium supplementation for 13 weeks significantly blocked a METH-induced increase in the lipid-peroxidation marker malondialdehyde (MDA), decreased the ratio of GSH/GSSG, and appeared to attenuate the loss of DA in the striatum and substantia nigra. Consistently, Imam et al. (1999) demonstrated that selenium pretreatment in drinking water for 1 week prevented (at least in part) the depletion of striatal DA induced by METH exposure in rodent brains. Further *in vitro* research supported their findings by showing that the increased oxidative stress induced by METH is reversed upon the treatment of SH-SY5Y neuronal cells with selenium, possibly through a reduction in glutathione peroxidase (GPx) levels. This effects is attributed to the incorporation of selenium into the amino acid selenocysteine by GPx1 and GPx4, which have antioxidant functions (Barayuga et al., 2013). However, in view of the narrow range between the therapeutic and toxic doses of selenium, as well as the dependence of the effect on the applied formulation, dose, and method of treatment, supplementation should be undertaken with appropriate precautions and avoidance of the side-effects of selenium (Ungvari et al., 2014; Ghosh et al., 2015; Kielczykowska et al., 2018).

Considering that mitochondrial dysfunction plays an important part in METH-induced neurotoxic insult, improvement of mitochondrial function might ameliorate the oxidative stress and neural damage associated with this disorder. The mood stabilizers lithium and valproate attenuate a series of METH-induced changes, such as reduction of mitochondrial Cyt c, the mitochondrial anti-apoptotic Bcl-2/Bax ratio, activity of mitochondrial Cyt c oxidase, and the expression of several proteins related to the apoptotic pathway. These phenomena illustrate that lithium and valproate enhance mitochondrial function and protect against the mitochondria-mediated toxicity of METH (Bachmann et al., 2009). Feier et al. (2013) found that lithium and valproate attenuated the effects of METH on the activity of enzymes in the Krebs cycle, thereby alleviating the impairment of respiratory chain complex activity (complexes I, II, III, and IV). In their study, the effects of lithium and valproate on some enzymes and in some brain areas were not always identical. For instance, interventions using lithium and valproate reversed the decrease in complex II activity in the hippocampus and striatum of rats; however, in the amygdala and prefrontal cortex, valproate (but not lithium) increased complex II activity, notably in the METH group. This region-specific effect on oxidative stress is in accordance with the previously shown heterogeneity of oxidative-stress parameters across brain regions and treatment regimens (Musavi and Kakkar, 2003).

Nicotinamide, which is a co-factor in the ETC, has been reported to be an efficacious treatment for mitochondrial encephalopathies, possibly through energy repletion (Penn et al., 1992). Huang et al. (1997) revealed that nicotinamide treatment before METH injection attenuated reductions in striatal DA and ATP content 5 days later *in vivo*. These findings suggest a close relationship between METH-induced perturbations of energy metabolism and dopaminergic neurotoxicity, and that potential therapeutic strategies might bypass bioenergetic defects if defective mitochondrial energy metabolism has a role in neurotoxicity. Inconsistently, Stephans et al. (1998) found that nicotinamide perfusion during METH administration had no effect on the long-term toxicity induced by METH. Given the localized effect of nicotinamide on nerve terminals (i.e., striatum), it is likely to produce consequences vastly different from those produced by systemic administration of METH. The distinct routes of administration in the two studies, as well as altered pharmacokinetics and bioavailability, might account for this discrepancy.

It has been reported that METH users experience a higher risk of developing Parkinson’s disease despite the different brain areas affected by METH and Parkinson’s disease (Callaghan et al., 2010, 2012; Curtin et al., 2015). Talipexole is used as an antiparkinsonian agent in Japan. It was shown to react strongly with OH^-^, with similar reaction kinetics against METH neurotoxicity *in vitro* and *in vivo*, indicating the potency of the neuroprotective action of talipexole due to its scavenging of OH^-^ (Mizuno et al., 1993; Kondo et al., 1998). In addition, Kish et al. (2017) suggested that, in principle, the striatal DA deficiency caused by METH may be corrected by DA substitution if safety and patient selection could be resolved. We hope that these findings will prompt researchers to investigate the potential of antiparkinsonian drugs and therapies targeted at specific brain regions for amelioration of the effects of METH on the brain.

### Targeting Excitotoxicity

#### Excitatory Receptor Antagonists

As discussed above, the excitatory toxicity of METH is closely related to the high release of Glu, which activates NMDARs and GluRs, leading to the influx of excess Ca^2+^ and triggering a series of intracellular cascade reactions. Therefore, several drugs targeting these receptors have been explored in recent years.

For example, melatonin is a potent protector against oxidative damage and regulates the movement of free Ca^2+^ intracellularly in the CNS (Suwanjang et al., 2016; Xu et al., 2016). Using a melatonin preparation, Singhakumar et al. (2015) demonstrated that the suppression of neuronal nitrogen-activated protein kinase and alteration of the NMDAR subunits NR2A and NR2B induced by METH were attenuated in the hippocampus of mice. Ekthuwapranee et al. (2015) suggested that melatonin reduced the METH-elicited inflammation, autophagy, and death of hippocampal progenitor cells *in vitro*. These findings are of interest to clinicians as melatonin protects against mitochondrial dysfunction, apoptosis, and dopaminergic degeneration, which are considered to contribute to several psychosomatic manifestations in METH abusers.

*N*-acetylcysteine (NAC) is a pro-glutamatergic compound. It is of considerable interest because NAC attenuates the excitatory toxicity of Glu by standardizing extracellular Glu levels in the nucleus accumbens via stimulation of the cystine–glutamate antiporter (Mcketin et al., 2017). Other actions of NAC include antioxidant activity, modulation of DA release, improvement of mitochondrial dysfunction, and reductions in levels of pro-inflammatory cytokines (Scofield and Kalivas, 2014). Several investigators have examined the effects of NAC in METH-exposure models owing to its broad efficacy against neuropsychiatric disorders involving schizophrenia, bipolar disorder, depression, memory impairments, and cognitive sequelae (Berk et al., 2012, 2014; Rapado-Castro et al., 2017). They found that pre-treatment with NAC significantly improved the reduction in density of DA transporters induced by high-dose METH treatment in mice, rats, and monkeys using PET (Hashimoto et al., 2004; Zhang et al., 2012). These results suggest that NAC reduces neurotoxic damage and possibly alleviates associated neuropsychiatric symptoms in METH abusers; these data were supported by clinical findings (Gray et al., 2012; Prado et al., 2015). Nonetheless, full-scale clinical trials to establish definitively if NAC has a therapeutic benefit (and the nature of this benefit) are needed.

Other chemical compounds targeting excitatory receptors have also shown therapeutic potential against METH. Baldwin et al. (1993) found that dizocilpine, a non-competitive NMDAR antagonist, acted as a potent anti-convulsant and protected dopaminergic neurons in the striatum against METH-induced neurotoxicity *in vivo*. Ma et al. (2013) reported that topiramate showed considerable potential in reducing the excitatory toxicity of METH. They hypothesized that topiramate has a complex mechanism of action that includes antagonism of several Glu receptors, blockade of voltage-dependent Na^+^ channels, and inhibition of carbonic anhydrase (Ma et al., 2013). In addition, Baptista et al. (2012) suggested that neuropeptide Y, with receptors Y1, Y2, and Y5, reduce Glu release to protect neurons from METH excitotoxicity. Activation of Y1 or Y2 receptors prevents METH-induced cell death, and the Y1 subtype is responsible for blocking the decrease in neuronal differentiation induced by METH (Baptista et al., 2012).

### Neuronal (n)NOS Inhibitors

The excitability of METH has been shown to be closely related to ONOO^-^ generation, which can be protected selectively by nNOS inhibitors, ONOO^-^ scavengers, or decomposition catalysts (Imam et al., 2000). nNOS inhibitors, such as 7-nitroindazole (7-NI) (Di Monte et al., 1996; Virmani et al., 2003), AR-R17477AR (Sanchez et al., 2003), S-Methylthiocitrulline, 3-bromo-7-nitroindazole (Itzhak et al., 2000), and the new nitrone-based radical scavenger S34176 (Lockhart et al., 2005), have shown significant protective effects and been used in investigations of METH-evoked neurotoxicity. Callahan and Ricaurte (1998) found that 7-NI generated hypothermic effects and afforded total protection against the DA depletions elicited by METH in the striatum of mice at 20°C. However, at 28°C, 7-NI produced minimal effects on body temperature and failed to alleviate METH-induced DA reductions. This findings suggest that the neuroprotective action of 7-NI was likely related to its ability to induce hypothermia (Callahan and Ricaurte, 1998). Itzhak et al. (2000) revealed that pretreatment with 7-NI before METH injection afforded protection against the depletion of dopaminergic markers induced by METH, but did not affect persistent hyperthermia at a low dose. These data suggest that diminished production of NO by nNOS inhibitors, rather than thermoregulation, might prevent METH-produced neurotoxicity (Itzhak et al., 2000). However, the detailed mechanism of action, such as the possible role of NO regulation by 7-NI, has not been elucidated.

### Anti-neuroinflammation

The inflammatory response induced by activated microglia plays a crucial role in the neurotoxicity of METH. Hence, blockade of microglial activation seems to be a promising method for the suppression of METH-induced neurotoxic effects (Abdul Muneer et al., 2011). The antibiotic minocycline has anti-inflammatory and neuroprotective effects in the CNS that are thought to be mediated by the inhibition of microglial activation (Zhang et al., 2006). Zhang et al. (2006) observed that the reduction of DA and DAT immunoreactivity after repeated administration of METH was attenuated dose-dependently if minocycline was used in the striatum of mice. They suggested that minocycline may (at least in part) protect against METH-induced neurotoxicity via inhibition of microglial activation *in vivo* (Zhang et al., 2006). The phosphodiesterase-4 inhibitor ibudilast increases brain levels of glial-derived neurotrophic factor and reduces microglial activation and production of pro-inflammatory cytokines (Charntikov et al., 2015). Clinical trials have shown that ibudilast can improve METH-induced acute injury (Bates et al., 2006). Further, ibudilast reverses the decrease in levels of synaptic signaling protein produced by chronic intake of METH (Charntikov et al., 2015). Although further clinical trials and animal experiments are needed, minocycline and ibudilast may have therapeutic potential for METH neurotoxicity through modulation of neuroimmune signaling. In addition, Raineri et al. (2012) revealed that modafinil (a cognitive enhancer with weak stimulant-like behavior used for the treatment of sleep apnea, narcolepsy, and shift work-induced sleep disorders) prevented METH-induced microglial and astroglial activation in the human brain, thus avoiding the induction of inflammatory processes.

### Vaccine Immunotherapies

Immunotherapies reduce the amount of drug in the CNS by triggering the production of antibodies binding the drug molecule after systemic absorption of METH (Ballester et al., 2017). Miller et al. (2013) found that, in rats receiving a KLH-conjugated METH-like hapten vaccine (MH6-KLH) and the vaccine succinyl MA (SMA–KLH), higher antibody titer-dependent METH serum concentrations, yet lower METH concentrations, in the CNS were observed, which suggested reduced METH concentrations in the brain. In addition, it was shown that anti-METH/AMP mAb4G9 therapy protected maternal and fetal rat brains from METH-induced damage (Gentry et al., 2006; White et al., 2014). Another study reported that a human–mouse monoclonal antibody binding to methylphenidate, named anti-METH antibody (mAB7F9), elicited a significant reduction in the METH concentration (Ballester et al., 2017). In particular, a combined approach using monoclonal and polyclonal antibodies was more effective in reducing the METH concentration in the brain (Hambuchen et al., 2015). However, the limitations of a vaccine remain; these include incomplete blockade of drug effects, prolonged delay in the production of sufficient circulating antibodies, and considerable variation in the antibody titer (Baracz and Cornish, 2016). Furthermore, given that anti-METH antibodies cannot cross the blood–brain barrier, this therapy may be too expensive because antibodies must be administered repeatedly to maintain an effective level (Chen et al., 2013).

### Drugs for Chronic Damage

Long-term exposure to METH leads to DA depletion, impairment of dopaminergic markers, and neuronal degeneration (Barr et al., 2002; Ballester et al., 2017). Therefore, extensive investigations, including those of several drugs in clinical trials, have been performed to explore efficacious pharmaceutical interventions for recovery from neural injury resulting from METH use. For instance, Reiner et al. (2014) reported that 9 *cis*-retinoic acid (9cRA), a biologically active derivative of Vit.A, reversed the METH-induced decrease in immunoreactivity and apoptosis of T-helper cells via inhibition of the export of nuclear receptor 77 from the nucleus to the cytosol in primary cultured dopaminergic neurons. Their *in vivo* experiments showed that 9cRA (delivered via the intracerebroventricular route) also antagonized the immunoreactivity of T-helper cells and locomotor activity in the striatum of rats indirectly through a signaling mechanism involving bone morphogenetic protein. This result is similar to that of a study by Yin et al. (2012) showing that 9cRA produced neuroprotective effects in a rodent model of Parkinson’s disease involving reduced rotational behavior and loss of T-helper cells in the substantia nigra, but increased DA release in the striatum. In the report by Yin et al. (2012), 9cRA was repeatedly administered through the intra-nasal route as intracerebral delivery is not feasible for repeated drug administration and may require chronic cannulation, whereas small molecules bypass the blood–brain barrier and reach brain parenchyma non-invasively if delivered via the intranasal route.

Cognition-enhancing as well as antidepressant and neuroprotective effects are conferred by 7, 8-Dihydroxyflavone (7, 8-DHF), a high-affinity tropomyosin receptor kinase B (TrkB) agonist that activates downstream signaling (Devi and Ohno, 2012; Hashimoto, 2013). Ren et al. (2014) observed that 7, 8-DHF significantly prevented the reduction of DAT and microglial activation in the striatum of mice after repeated METH administration. In their study, it was investigated whether 7, 8-DHF prevents neurotoxicity through a signaling pathway upstream of DA terminals, and if reduction of microglial activation occurs as a consequence (rather than a cause) of METH-induced neurotoxicity. In view of the vital role of microglial activation in neurotoxicity, this should be explored in further detail. Endogenous hormones have shown therapeutic potential in METH treatment. For instance, Cholecystokinin-8 (CCK-8) pretreatment has been shown to attenuate the decrease in expression of TH and DAT in the striatum (Gou et al., 2015). Overall, these drugs have shown the potential for amelioration of hypo-dopaminergic status and neuron degeneration; however, their precise mechanism of action is not known (**Table 2**).

**Table 2:** The summary of pharmacotherapy approaches in METH-induced neurotoxicity *in vivo* or *in vitro.*

Agent	Mechanism of action	Species	METH dosing regimen	Key results	Reference
Vit. C	A scavenger of free radicals	Rat cortical neuron-glia cultures	5 mM for 1, 3, 6, 12, 18, and 24 h	• Attenuated METH-induced ROS production	Huang et al., 2012
Selenium	An antioxidant	Mice	10 mg/kg i.p.×4, at 2 h. intervals	• Attenuated METH-induced DA depletion	Imam et al., 1999; Kim et al., 1999
				• Attenuated METH-induced reductions in GSH level, GSH/GSSG ratio, and depletion of DA	
Selenium	An antioxidant	SH-SY5Y cells	100 mM for 24 h	• Increased the GPx1 and GPx4 proteins levels	Barayuga et al., 2013
				• Limited METH-induced ROS production	
Lithium/valproate	Regulation of Cyt c, Bcl-2/Bax ratio, and apoptosis proteins	Rats	No exact dose	• Attenuated METH-induced decreases in mitochondrial Cyt c and Bcl-2/Bax ratio Inhibited the METH-induced reduction of COX activity	Bachmann et al., 2009
Talipexole	(OH^-^)-scavenging and D2 agonist activity	Mice	5mg/kg i.p.×4, at 2 h. intervals	• Attenuated METH-induced reduction of TH activity	Kondo et al., 1998
Melatonin	An antioxidant and regulates free calcium ions movement intracellularly	Hippocampal neural progenitor cells	Concentration range: 50, 100, 300, 500, 600, 800, and 1000 μM for 3 days	• Ameliorated METH-induced decrease in proliferation	Ekthuwapranee et al., 2015
Dizocilpine	A non-competitive NMDA antagonist	Rats	15 mg/kg i.p.×4, at 2 h. intervals	• Provided substantial protection against neurotoxic loss of striatal DA and 5-HT	Baldwin et al., 1993
NAC	Stimulates the cystine–glutamate antiporter	Mice	1 mg/kg/day, s.c. for 7 days	• Suppressed METH-induced elevation of extracellular DA levels	Miyamoto et al., 2014
Topiramate	Antagonism of several GluRs, blockade of voltage-dependent sodium channels	volunteers	No exact dose	• Increased GABA activity, antagonism of several GluRs	Ballester et al., 2017
Neuropeptide Y	Antagonism of several GluRs, blockade of voltage-dependent sodium channels	neurosphere cultures	10 nM for 24 h	• Prevented METH-induced apoptosis and decrease of neuronal differentiation	Baptista et al., 2012
7-NI	A potent inhibitor of nNOS	Mice	10 mg/kg i.p. ×4, at 2 h intervals	• Protected against METH-induced DA depletion	Di Monte et al., 1996
				• Counteracted the decrease in the DA metabolite level	
AR-R17477AR	nNOS inhibitor	Mice	1, 3, 6 and 9 mg/kg, i.p. ×3, at 3 h intervals	• Attenuated the decrease in striatal DA and DA metabolite concentrations	Sanchez et al., 2003
S-methylthiocitrulline	nNOS inhibitor	Mice	5 mg/kg i.p. ×3, at 3 h intervals	• Protection against the depletion of dopaminergic markers	Itzhak et al., 2000
3-bromo-7-nitroindazole	nNOS inhibitor	Mice	5 mg/kg i.p. ×3, at 3 h intervals	• Afforded protection against the depletion of dopaminergic markers	Itzhak et al., 2000
S34176	Nitrone-based radical scavenger	Mice	5 mg/kg i.p. ×4, at 2 h intervals	• Attenuated METH-mediated depletion of striatal DA levels	Lockhart et al., 2005
Minocycline	An antibiotic	Mice	3 mg/kg/day s.c., once daily for 5 days or 3 mg/kg s.c. ×3, at 3 h intervals	• Attenuated the level of DA and its major metabolite, 3,4-dihydroxyphenyl acetic acid	Zhang et al., 2006
				• Attenuated a reduction in DAT immunoreactivity	
Ibudilast	Phosphodiesterase-4 inhibitor	Rats	Self-administered 0.05 mg/kg/infusion for 25 days	• Attenuated METH-seeking during abstinence	Charntikov et al., 2015
Modafinil	A cognitive enhancer	Mice	5 mg/kg, i.p. ×4, 2 h intervals	• Counteracted the decrease of TH and DAT levels Prevented METH-induced increases in BAX/Bcl-2 ratio	Raineri et al., 2012
MH6-KLH	METH vaccine	Rats	0, 1.0, 5.6 mg/kg, s.c., once	• Produced high antibody titers of METH and sequestered METH in the periphery of rats	Miller et al., 2013
				• Blocked the thermoregulatory and psychomotor responses produced by METH	
SMA–KLH	METH vaccine	Mice	1, 2, or 3 mg/kg i.p., once	• Reduced METH-induced conditioned approach behaviors	Shen et al., 2013
				• Decreased conditioned activity levels	
Anti-METH mAb4G9	Anti-METH antibody	Rats	1 mg/kg i.v., once	• Reduced METH brain values	White et al., 2014
9cRA	A active derivative of vitamin A	Rats	5 mg/kg, s.c. ×4, 2 h intervals	• Reversed METH-induced TH immunoreactivity, and neurodegeneration in dopaminergic neurons	Reiner et al., 2014
7, 8-DHF	A novel potent TrkB agonist	Mice	3.0 mg/kg/day s.c., once daily for 5 days	• Attenuated the reduction of DAT in the striatum	Ren et al., 2014
				• Attenuated microglial activation in the striatum	
CCK-8	An endogenous hormone	Mice	Concentration range: 0, 3, 10, 20, and 40 mg/kg, i.p. ×4, 3 h intervals	• Attenuated METH-induced hyperthermia, the decrease of TH and DAT in the striatum, and TH in the substantial Ingra	Gou et al., 2015


*i.p., intraperitoneal; i.v., intravenous; s.c., subcutaneous; ROS, reactive oxygen species; DAT, dopamine transporter; METH, Methamphetamine; Cyt c, cytochrome c.*

## Future Perspectives

The complex mechanisms underlying METH-evoked neurotoxicity affect sub-regions of the CNS in various ways. Important advances have been made regarding the basic neurobiology of METH-produced neurotoxicity; however, these findings have not resulted in the development of efficacious drug therapy. Nevertheless, in view of the great harm elicited by METH to the CNS, exploration of toxic mechanisms and pharmacotherapies are anticipated in preclinical/clinical investigations. Natural compounds such as 1-methyl-l, 2, 3, 4-tetrahydroisoquinoline (1MeTIQ), resveratrol, curcumin, and gingko biloba have garnered considerable inhibition owing to their ability to scavenge excess free radicals to protect neurons. Accordingly, some researchers have shifted focus to natural compounds to make major breakthroughs. Wasik and Antkiewicz-Michaluk (2017) suggested that 1MeTIQ can apply a brake to several processes related to neurotoxicity (e.g., elimination of free radicals, inhibition of MAO, and Glu-dependent excitotoxicity) without side-effects. Therefore, the use of natural compounds may represent a promising strategy for preventing METH neurotoxicity. In a recent study, the therapeutic effect of carbon nanotubes, which enabled oxidation of METH-enhanced extracellular DA in the striatum of mice, was demonstrated (Xue et al., 2016). Considering the favorable properties (e.g., large surface areas, superior bundle strength, and highly electrostatic attraction to neurotransmitters such as DA) of nanotubes, their use merits further research as a new approach for treatment of METH abuse-induced neurotoxicity.

## Author Contributions

XY and JY wrote the manuscript. YW, QL, YZ, LC, and YD completed the figures and the tables. LL, KX, and C-xY provided advice. All authors reviewed the manuscript.

## Conflict of Interest Statement

The authors declare that the research was conducted in the absence of any commercial or financial relationships that could be construed as a potential conflict of interest.
